# Dominant factors of the phosphorus regulatory network differ under various dietary phosphate loads in healthy individuals

**DOI:** 10.1080/0886022X.2021.1945463

**Published:** 2021-06-30

**Authors:** Guoxin Ye, Jiaying Zhang, Zhaori Bi, Weichen Zhang, Minmin Zhang, Qian Zhang, Mengjing Wang, Jing Chen

**Affiliations:** a Nephrology, Huashan Hospital, Fudan University, Shanghai, China; b Division of Nutrition, Huashan Hospital, Fudan University, Shanghai, China; c National Clinical Research Center for Aging and Medicine, Huashan Hospital, Fudan University, Shanghai, China

**Keywords:** Phosphorus regulation network, granger causality, phosphatonins, dietary phosphorus

## Abstract

**Background:**

The purpose of this study was to explore the contribution of each factor of the phosphorus metabolism network following phosphorus diet intervention *via* Granger causality analysis.

**Methods:**

In this study, a total of six healthy male volunteers were enrolled. All participants sequentially received regular, low-, and high-phosphorus diets. Consumption of each diet lasted for five days, with a 5-day washout period between different diets. Blood and urinary samples were collected on the fifth day of consumption of each diet at 9 time points (00:00, 04:00, 08:00, 10:00, 12:00, 14:00, 16:00, 20:00, 24:00) for measurements of serum levels of phosphate, calcium, PTH, FGF23, BALP, α-Klotho, and 1,25 D and urinary phosphorus excretion. Granger causality and the centrality of the above variables in the phosphorus network were analyzed by pairwise panel Granger causality analysis using the time-series data.

**Results:**

The mean age of the participants was 28.5 ± 2.1 years. By using Granger causality analysis, we found that the α-Klotho level had the strongest connection with and played a key role in influencing the other variables. In addition, urinary phosphorus excretion was frequently regulated by other variables in the network of phosphorus metabolism following a regular phosphorus diet. After low-phosphorus diet intervention, serum phosphate affected the other factors the most, and the 1,25 D level was the main outcome factor, while urinary phosphorus excretion was the most strongly associated variable in the network of phosphorus metabolism. After high-phosphorus diet intervention, FGF23 and 1,25 D played a more critical role in active regulation and passive regulation in the Granger causality analysis.

**Conclusions:**

Variations in dietary phosphorus intake led to changes in the central factors involved in phosphorus metabolism.

## Introduction

Inorganic phosphorus is an essential element for intracellular signal transduction, energy production, and the formation of the skeletal extracellular matrix [1,2]. Therefore, the maintenance of phosphorus homeostasis is of great importance. Phosphorus homeostasis is regulated by a complex network consisting of many regulatory factors and several organs, including the intestine, kidney, and bone. Phosphorus is first absorbed in the intestine with an absorption rate of approximately 65–70%. Excretion of phosphorus takes place primarily in the kidney. The exchange of phosphorus between extracellular and bone storage pools also plays an essential role in phosphorus homeostasis.

Various regulatory factors are involved in regulating the above process. Parathyroid hormone (PTH) and fibroblast growth factor 23 (FGF23) are known to promote phosphorus excretion from the kidney. 1,25(OH)_2_D_3_ (1,25 D) promotes the intestinal transfer and kidney reabsorption of phosphorus [3]. α-Klotho is an aging-related protein that combines with the FGF receptor (FGFR) to form a coreceptor that mediates the biological function of FGF23 [3]. Recent studies suggest that bone-specific alkaline phosphatase (BALP) is involved in the regulation of bone metabolism through modulation of the balance between inorganic phosphate and inorganic pyrophosphate [4]. In addition, serum calcium is negatively associated with serum phosphate. These regulatory factors interact with each other to avoid the amplification of a single effect [5–7] and maintain phosphorus homeostasis. Previous studies reported the change in the levels of single hormones separately without exploring the interactions among these factors. However, under a load of dietary phosphorus, the contribution of the key factors in the phosphorus regulatory network is still controversial [8–10].

In this study, we applied Granger causality analysis to establish a phosphorus metabolism network in response to three types of dietary interventions and showed Granger causality among the regulatory factors. This study provides potential causal inference relationships and a meaningful hypotheses for future empirical research.

## Methods

### Study population

This study recruited six healthy male volunteers. The inclusion criteria were as follows: (1) good health and male sex; (2) an age of 18–45 years; (3) normal electrocardiography, chest radiography, and biochemical indicators; and (4) no previous disease history. Subjects were excluded if they (1) were mentally or physically disabled; (2) reported a history of alcohol abuse; or (3) participated in other clinical trials within one month. The Human Ethics Committee of Huashan Hospital, Fudan University, Shanghai, China, approved the present study (Protocol Number: KY2015-262). This phase I clinical trial was registered at ClinicalTrials.gov (#NCT03208075, 5 July 2017). Written informed consent was obtained from all participants prior to the study.

### Study design

All participants sequentially received the following three different diets at the Phase I Clinical Research Center of Huashan Hospital: (1) a regular phosphorus diet (Pi: 1500 mg/day), (2) a low-phosphorus diet (Pi: 500 mg/day), and (3) a high-phosphorus diet (Pi: 2300 mg/day). Each dietary intervention lasted for five days, with a 5-day washout period between the different diets (Supplemental Figure 1). The levels of sodium, calcium, and calories were consistent among these diets (Supplemental Table 1). The phosphorus in each diet was from the same source. Meals were given at 7:30, 11:30, and 17:30. Participants were allowed to engage in light physical activity in the ward but not outdoors. On the first day of each diet intervention, blood and urine samples were collected at 4:00 and were used as baseline measurements. On the fifth day of each diet intervention, blood and urinary samples were collected from each subject at 9 time points with symmetric time intervals (00:00, 04:00, 08:00, 10:00, 12:00, 14:00, 16:00, 20:00, 24:00) for measurements of serum levels of phosphate, calcium, PTH, FGF23, BALP, α-Klotho, and 1,25 D and urinary phosphorus excretion. The symmetrical sampling time interval was beneficial for establishing stationary time-series data models.

### Measurements

Serum and urine levels of biochemical variables were measured with an automatic biochemical analyzer (HITACHI 7600-020), including serum phosphate, calcium, albumin, serum glucose, triglycerides, total cholesterol, glutamic transferase, aspartate transferase, urea nitrogen, serum creatinine, and uric acid. Serum levels of PTH, FGF23, α-Klotho, 1,25 D and BALP were assessed by ELISA kits (Immutopics, San Clemente, CA; IBL, MN, USA; Immunodiagnostic System, Boldon, UK). The calcium correction formula, i.e., serum corrected calcium (mmol/L) = total serum calcium (mmol/L) + 0.02 [40 (g/L) – albumin (g/L)], was applied for the estimation of serum calcium levels.

### Mathematical model

Granger causality is a statistical test used to determine whether one time series can be used to predict another. The time series X is said to Granger-cause Y if the T-tests or F-tests on the lag value of X and Y are statistically significant, which indicates that the X value ​​provides information for the future value of Y. Panel Granger causality [11,12], which emphasizes the causality among multi-individual data involving measure variables, is an extension of Granger causality analysis [13]. We prefer it for three keys. (1) It is suitable for time-series data. Bayesian-based methods cannot conveniently analyze time-series data because they must satisfy the assumptions of causal sufficiency, faithfulness, and causal Markov conditions must be satisfied [14]. (2) It allows us to analyze panel data. The format of our experimental data is naturally a panel format that contains multiple subjects who consumed varying amounts of dietary phosphorus in the designed time slots. (3) It supports the quantification of causality [15]. This feature enables us to present the potential regulatory relationships between variables *via* a weighted adjacency matrix.

To establish the panel Granger causality model, we first organized the experimental data in the form of a panel and ensured that all data were time-stationary *via* the Levin, Lin and Chu (LLC) panel data unit root test method [16]. Then, pairwise panel Granger causality [12] analysis was performed to obtain the weighted adjacency matrix and to construct a graph of the relationships between variables. Finally, we applied graph analysis to quantify causality. In the graph, betweenness centrality is a measure of the connectedness of a vertex. It identifies the index that plays a ‘bridge spanning’ role in a network. Indegree centrality is calculated as the number of edges pointing to a vertex $x_{i}$ divided by the number of all possible paths. Outdegree centrality is calculated as the number of edges pointing out from a vertex $x_{i}$ divided by the number of all possible paths. Density indicates the complexity of a given graph G. The mathematical details of the panel Granger causality method are elaborated in the Supplemental Methods.

The causality inferences in this study were all derived from the Granger causality analysis of the experimental data. The deterministic causality needs to be verified by further random experiments based on the inferred relationship; thus, the causal conclusions described in this study do not necessarily constitute factual causality. The aim of our study is to provide potential causal inference relationships and meaningful hypotheses for future empirical research.

### Statistics

All data are expressed as the means ± SEMs unless otherwise specified. The Kruskal-Wallis test followed by Bonferroni correction was used for intergroup comparisons. Repeated measures correlation analysis was used to estimate the common linear association in paired measures data of phosphorus metabolism [17]. The panel data unit root test and Granger causality analysis were performed using the STATA packages xtunitroot llc [16] and xtgranger [18]. Graph construction and analysis were performed with the Python package NetworkX [19]. A *p*-value of less than 0.05 was considered statistically significant.

## Results

### Participant characteristics

The baseline characteristics of the six healthy male volunteers are summarized in Table 1.

**Table 1.** Baseline characteristics of six healthy participants.

Characteristics (Male, *n* = 6)	Mean ± SEM
Age (year)	28.5 ± 2.1
Weight (kg)	64.5 ± 2.4
Height (cm)	173.7 ± 2.2
Systolic pressure (mmHg)	111.8 ± 5.2
Diastolic pressure (mmHg)	75.3 ± 3.1
Body mass index (kg/m^2^)	21.4 ± 0.5
eGFR (mL/min per 1.73m^2^)	114.7 ± 2.9
Hemoglobin (g/L)	141.8 ± 4.5
Serum albumin (g/L)	47.0 ± 0.9
Blood biochemical indicators	
Glucose (mmol/L)	4.8 ± 0.2
TG (mmol/L)	0.6 ± 0.1
TC (mmol/L)	4.0 ± 0.3
ALT (U/L)	17.3 ± 1.9
AST (U/L)	16.7 ± 0.7
BUN (mmol/L)	4.0 ± 0.3
Serum creatinine (umol/L)	78.3 ± 2.5
Uric acid (mmol/L)	0.3 ± 0.1
Serum phosphate (mmol/L)	1.1 ± 0.1
Serum calcium (mmol/L)	2.1 ± 0.1

Data are shown as mean ± SEM or median (interquartile range). Abbreviations: eGFR, estimated glomerular filtration rate calculated by the EPI equation; TG: triglyceride; TC: total cholesterol; ALT: glutamic-pyruvic transaminase; AST: glutamic-oxalacetic transaminase; BUN: urea nitrogen; Serum calcium, serum corrected calcium. Conversion factors for units: triglycerides in mmol/L to mg/dL, 88.6; serum creatinine in umol/L to mg/dL, 0.01131; eGFR in mL/min/1.73m^2^ to mL/s/1.73m^2^, 0.01667; serum calcium in mmol/L to mg/dL, 4; serum phosphate in mmol/L to mg/dL, 3.1.

### Time course of the change in the levels of mineral metabolism-related variables

**Figure 1.**
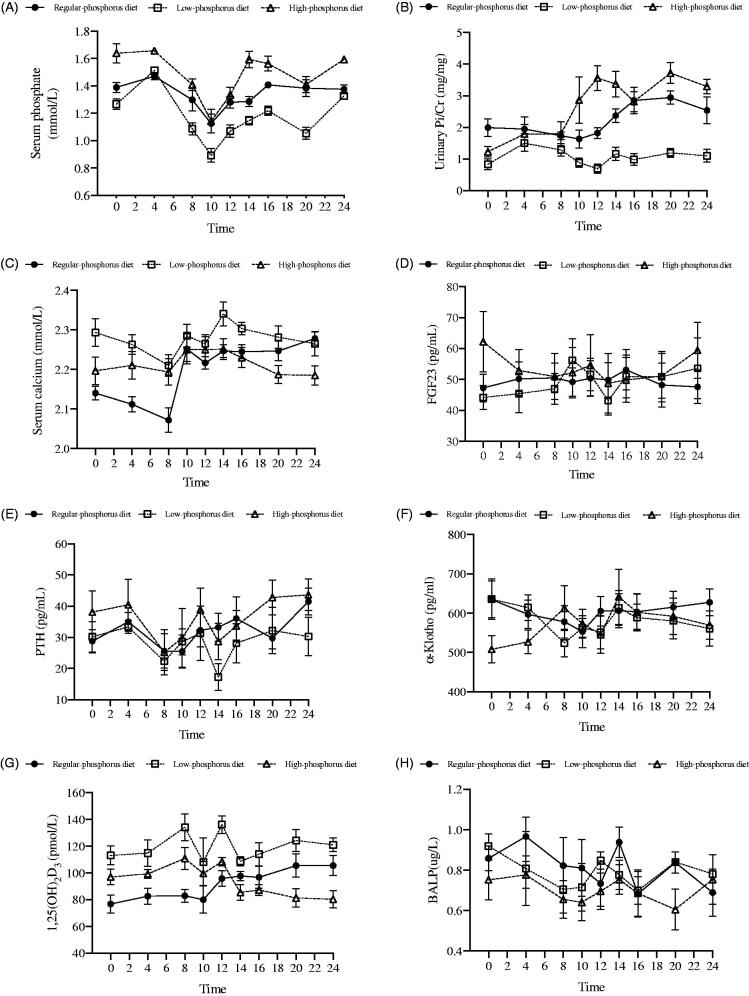
Time courses of changes in the levels of mineral metabolism-related variables after each diet intervention. Changes in serum phosphate (A), urinary Pi/Cr (B), serum calcium (C), FGF23 (D), PTH (E), α-Klotho (F), 1,25(OH)_2_D_3_ (G) and BALP (H) levels in six healthy participants at 9 times points during the last 24 hours of a regular phosphorus diet, low-phosphorus diet, and high-phosphorus diet intervention. Urinary Pi/Cr: urinary phosphorus/creatinine rate; FGF23: fibroblast growth factor 23; PTH: parathyroid hormone; 1,25(OH)_2_D_3_: 1,25-dihydroxyvitamin D3; BALP: bone alkaline phosphatase.

Figure 1 shows the time course of changes in the levels of serum phosphate, serum calcium, PTH, 1,25 D, BALP, α-Klotho, and FGF23 and urinary phosphorus excretion during the last 24 h.

### Nutritional status and mineral metabolism before and after diet interventions

Intergroup comparisons showed that nutritional status and mineral metabolism before each diet intervention did not differ (Supplemental Table 2). However, compared with the regular phosphorus diet, the low-phosphorus diet decreased serum phosphate levels and urinary phosphorus excretion but significantly increased serum concentrations of calcium and 1,25 D. In contrast, compared with the regular and low-phosphorus diets, the high-phosphorus diet increased serum phosphate levels and urinary phosphorus excretion but significantly decreased the concentration of BALP. In addition, compared with the low-phosphorus diet, the high-phosphorus diet decreased serum 1,25 D levels (Supplemental Table 3).

### Repeated measures correlation analysis

In the regular-phosphorus diet intervention, the serum contents of PTH were correlated with serum phosphate and BALP levels and urinary phosphorus excretion. Serum 1,25 D levels correlated with serum calcium levels and urinary phosphorus excretion. In the low-phosphorus diet intervention, the level of urinary phosphorus excretion was associated with the serum phosphate concentration. The serum calcium concentration correlated with the serum 1,25 D level. Serum α-Klotho was associated with serum levels of FGF23 and 1,25 D. After the high-phosphorus diet intervention, serum calcium levels correlated with serum α-Klotho and FGF23 levels. The level of 1,25 D correlated with the serum phosphate concentration and urinary phosphorus excretion. Serum α-Klotho levels were associated with FGF23 and PTH levels (Table 2).

**Table 2.** Repeated measures correlation analysis of each mineral metabolic variable following different types of phosphorus diets.

Gray fill area: Repeated measures correlation coefficient between two metabolic variables; white fill area: *p*-value for the correlation of two metabolic variables. Abbreviations: Serum Ca, serum corrected calcium; Serum Pi: serum phosphate; FGF23: fibroblast growth factor 23; BALP: bone alkaline phosphatase; PTH: parathyroid hormone; Urinary Pi/Cr: urinary phosphorus/creatinine rate.

### Granger causality and graph analysis

**Figure 2.**
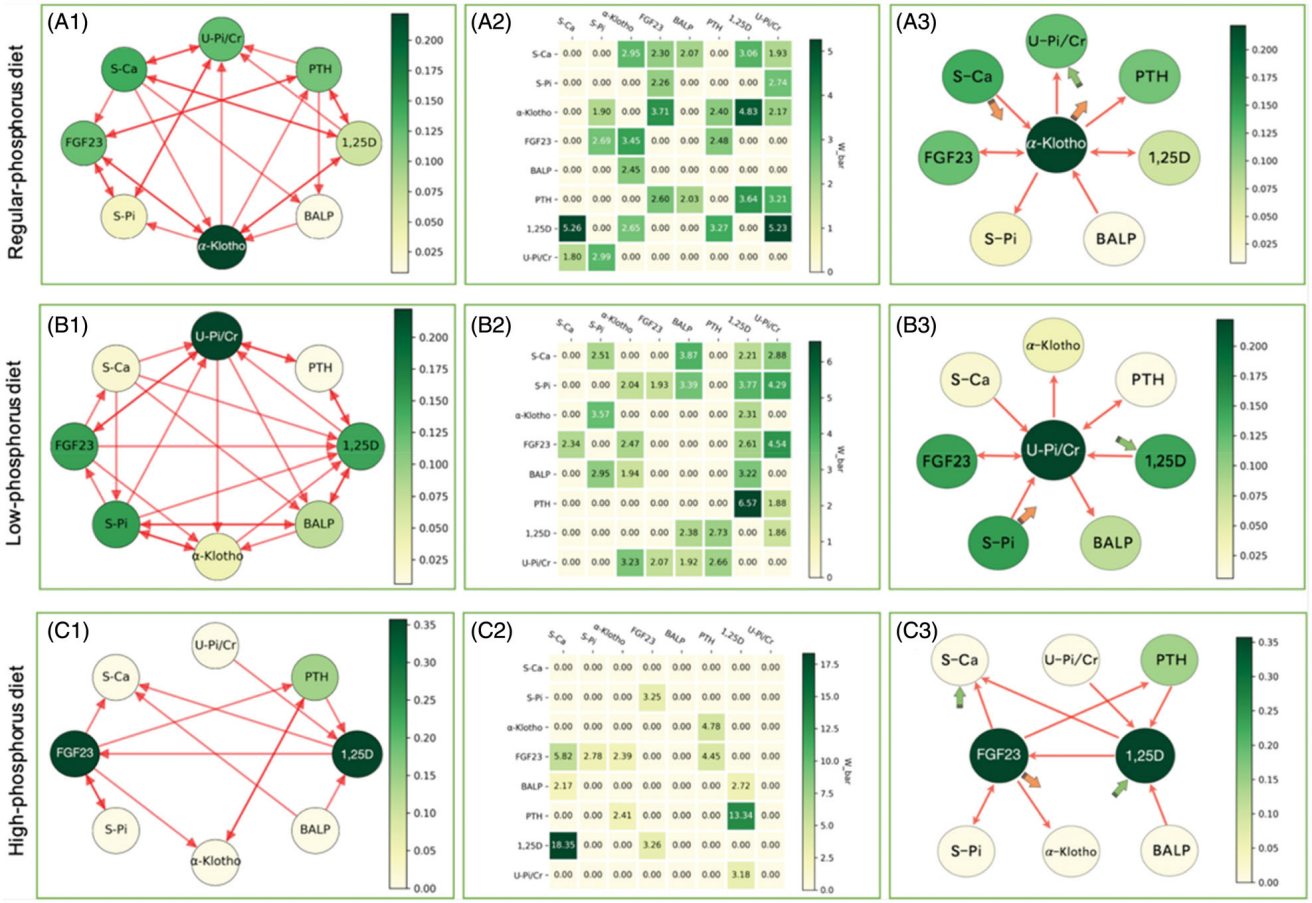
The network of phosphorus metabolism according to Granger causality analysis following regular, low-, and high-phosphorus diet interventions. Causality graph with the betweenness centrality index (A1, B1 and C1), the weighted adjacency matrices (A2, B2 and C2) and the highest betweenness and degree effects (A3, B3 and C3) following regular, low- and high-phosphorus diet interventions. The shading, from light green for the lowest value to dark green for the highest value, represents the values of the variables (betweenness in the nodes of graphs A1 and A3, weighted adjacency in matrix A2). The shading scale is to the right of each panel. Graph A3 is the simplified version of graph A1, in which the phosphatonin with the highest betweenness value is placed in the middle, and the thin arrows show the regulatory relationships associated with this phosphatonin. In addition, the large orange and green arrows indicate the variables with the highest outdegree and indegree values. Abbreviations: U-Pi/Cr, urinary phosphorus/creatinine rate; PTH, parathyroid hormone; FGF23, fibroblast growth factor 23; BALP, bone alkaline phosphatase; 1,25D, 1,25-dihydroxyvitamin D3; S-Pi, serum phosphate; S-Ca, serum corrected calcium.

The network of phosphorus metabolism constructed using Granger causality analysis is shown in Figure 2. In A1, B1 and C1, each node represents a mineral metabolic variable. The arrows indicate connections between the two variables, in which the head of the arrow represents the effect and the tail represents the cause. The importance (betweenness centrality) of a node is indicated by shading. The darker the shade is, the greater the chance that the variable tended to regulate or be regulated by other factors. In A2, B2, and C2, the Granger causal relationship is represented by the weighted adjacency matrix; the larger the value is, the stronger the Granger causality. A value of 0 indicates no Granger causal relationship. In Table 3, the regulatory relationships are represented by indegree/outdegree values, where variables with the highest outdegree value have the strongest regulatory effect on other variables involved in phosphorus metabolism. Variables with the highest indegree value were those that were the most regulated. In A3, B3 and C3, we selected variables with the top importance ranks (betweenness centrality) in the center of the graph and showed the causal connections associated with these variables. The large orange and green arrows indicate variables with the most significant regulatory effects (highest outdegree and indegree values).

**Table 3.** Graph theory and network analysis of the interactions among variables in phosphorus regulatory network following different dietary interventions.

	1,25(OH)_2_D_3_	Serum Pi	FGF23	Serum Ca	Urinary Pi/Cr	α-Klotho	BALP	PTH
Regular-phosphorus diet								
Betweenness	0.07	0.03	0.12	0.14	0.13	0.22	0.01	0.12
Out degree	0.57	0.29	0.43	0.71	0.29	0.71	0.14	0.57
In degree	0.43	0.43	0.57	0.29	0.71	0.57	0.29	0.43
Low-phosphorus diet								
Betweenness	0.15	0.15	0.15	0.02	0.22	0.04	0.08	0.01
Out degree	0.43	0.71	0.57	0.57	0.57	0.29	0.43	0.29
In degree	0.86	0.43	0.29	0.14	0.71	0.57	0.57	0.29
High-phosphorus diet								
Betweenness	0.36	0	0.36	0	0	0	0	0.14
Out degree	0.29	0.14	0.57	0	0.14	0.14	0.29	0.29
In degree	0.43	0.14	0.29	0.43	0	0.29	0	0.29

Serum Ca: serum corrected calcium; Serum Pi: serum phosphate; FGF23: fibroblast growth factor 23; BALP: bone alkaline phosphatase; PTH: parathyroid hormone; Urinary Pi/Cr: urinary phosphorus/creatinine rate.

#### α-Klotho plays a Central role in phosphorus metabolism during a regular phosphorus diet consumption

During regular phosphorus diet consumption, α-Klotho played a central role in phosphorus metabolism with a betweenness of 0.22. α-Klotho and serum calcium also had the highest outdegree value of 0.71, and urinary phosphorus excretion had the highest indegree value of 0.71. From the causal graph, we observed that α-Klotho was causally related to urinary phosphorus excretion and serum PTH and serum phosphate levels, and it interacted with serum FGF23 and 1,25 D. In addition, α-Klotho was regulated by BALP and serum calcium (Figure 2 (A3)). In contrast, urinary phosphorus excretion was regulated by 1,25 D, PTH, and α-Klotho and interacted with serum calcium and phosphate (Figure 2 (A1)).

#### Urinary phosphorus excretion plays a Central role in phosphorus metabolism during low-phosphorus diet consumption

Following low-phosphorus diet consumption, urinary phosphorus excretion played a central role in phosphorus metabolism with a betweenness of 0.22. Serum phosphate had the highest outdegree value of 0.71, and 1,25 D had the highest indegree value of 0.86 (Figure 2 (B3)). From the causal graph, we could see that serum phosphate directly affected urinary phosphorus excretion and serum levels of 1,25 D and FGF23, and it interacted with BALP and α-Klotho. In addition, serum phosphate was regulated by serum calcium. In contrast, 1,25 D was regulated by serum calcium, FGF23, phosphate, and α-Klotho and interacted with PTH and BALP (Figure 2 (B1)).

#### 1,25D And FGF23 play a Central role in phosphorus metabolism during high-phosphorus diet consumption

During high-phosphorus diet intervention, 1,25 D and FGF23 played a central role in phosphorus metabolism, with a betweenness of 0.36. Serum FGF23 had the highest outdegree value of 0.57. Serum calcium and 1,25 D had the highest indegree value of 0.43. The causal graph showed that serum FGF23 regulated PTH, serum calcium, and α-Klotho, and it interacted with serum phosphate but was regulated by 1,25 D. In contrast, serum 1,25 D was regulated by PTH, urinary phosphorus excretion, and BALP. It also affected serum calcium and FGF23 levels (Figure 2 (C3)).

The density describes the complexity of the metabolic network. The densities of the network following regular, low- and high-phosphorus diet interventions were 0.46, 0.48, and 0.23, respectively.

## Discussion

In this study, we performed Granger causality analysis to establish networks of phosphorus metabolism in response to three types of dietary interventions and showed the centrality and Granger causal relationships among the regulatory factors. We found that dietary phosphorus intake contributed to changes in central regulatory factors in phosphorus metabolism. The key regulatory factors involved in phosphorus metabolism following regular, low- and high-phosphorus diet consumption were α-Klotho, urinary phosphorus excretion, and 1,25 D and FGF23, respectively. In addition, α-Klotho and serum calcium affected the other factors the most, and urinary phosphorus excretion was regulated by other variables, mostly during regular phosphorus diet consumption. Serum phosphate was the factor affecting other variables the most, and the 1,25 D level was the main outcome factor during the low-phosphorus diet. Serum FGF23 was the primary regulator of the other factors, and 1,25 D and serum calcium levels were the main outcome factors following high-phosphorus diet consumption.

Granger causality has been widely used in econometric studies [20]. It is a powerful method to establish complex function networks and detect causal relationships. Thus, it has also been widely used in neuroimaging and neurophysiology [21–23]. The network of phosphorus metabolism is similar to the neural network. However, *in vitro* studies have mainly reported mutual regulatory relationships between each pair of factors, such as the mutual regulation between FGF23 and 1,25 D [24,25], calcium and PTH, phosphate and PTH [26–28]. Previous *in vivo* studies in humans corroborate some of the above individual relationships in phosphorus metabolism after consumption of diets with different amounts of phosphorus [8,10]. However, these studies did not consider the regulation of phosphorus as a network, and inconsistent results were always reached.

Here, we are the first to find that α-Klotho is the variable with the strongest associations and plays a key role in influencing other variables in the network of phosphorus metabolism following consumption of a regular phosphorus diet. α-Klotho has been widely recognized as a ligand for FGFR in the FGF23-Klotho endocrine axis that mediates phosphorus excretion in the kidney [29]. It has also been found that α-Klotho directly increasing urinary phosphorus excretion by promoting endocytosis and degradation of NPT2a in the BBM of proximal tubules [3]. Soluble α-Klotho also inhibits the expression of housekeeping sodium phosphate cotransporters, such as Pit-1 and Pit-2 (Type III sodium phosphate cotransporters), which are involved in cellular phosphate uptake [30]. In addition, it has been reported that soluble α-Klotho induces a significant increase in FGF23 expression and PTH secretion but decreases 1,25 D production [31–35]. In contrast, FGF23 and 1,25 D can also reduce and induce α-Klotho expression, respectively [33,36–38]. Consistently, the Granger network in this study demonstrated that α-Klotho had a causal relationship with FGF23 and 1,25 D levels and urinary phosphorus excretion. Urinary phosphorus excretion showed the highest indegree value after the regular-phosphorus diet intervention, indicating that urinary phosphorus excretion was the most frequently regulated by other variables during phosphorus metabolism.

After the low-phosphorus diet intervention, serum phosphate affected the other factors the most, and the 1,25 D level was the main outcome factor. Additionally, urinary phosphorus excretion was the most strongly connected variable in the network of phosphorus metabolism. Previous evidence suggests that phosphorus exerts regulatory effects on hormone pathways. A decrease in serum phosphate levels suppresses the secretion of FGF23. In osteocyte-like cells, the mRNA expression of Cyp27b1 is upregulated by phosphorus in a dose-dependent manner [39], implying that phosphorus might have a direct impact on vitamin D metabolism. In the proximal tubules, changes in serum phosphate levels directly regulate urinary phosphorus excretion through targeting of NPT2a/2c. Combined with our results, these findings lead us to speculate that a low-phosphorus diet intervention may quickly induce a decrease in serum phosphate levels and trigger a series of hormonal regulatory and urinary phosphorus excretion reactions. Various regulatory factors suppress urinary phosphorus excretion by decreasing NPT2a/2c expression in proximal tubules and promote the absorption of phosphorus in the intestine by regulating the expression of 1,25 D and its downstream NPT2b [40,41], to maintain serum phosphate levels in the normal range.

Following the high-phosphorus diet intervention, FGF23 and 1,25 D played a critical role. In addition to increased urinary phosphorus excretion, which may be mediated by FGF23 and PTH, we speculate that bone metabolism and decreased intestinal absorption may be involved in phosphorus homeostasis since FGF23 is predominately expressed in osteocytes. Bone also contributes to the maintenance of serum phosphate as the largest exchangeable depot. A higher bone mineralization or lower bone absorption may contribute to decreasing serum phosphate levels. However, further research may be needed to verify this hypothesis [42]. In addition, FGF23 was also indicated to indirectly decrease the intestinal absorption of phosphorus by inhibiting the production of 1,25 D [43]. 1,25 D also plays a vital role in keeping the serum phosphate level within a normal range and regulating the production of FGF23 through vitamin D receptors and vitamin D response elements [6]. Therefore, more attention needs to be paid to the levels of FGF23 and 1,25 D to maintain phosphorus homeostasis following a high-phosphorus diet intervention.

In addition, serum calcium affected the other factors the most during consumption of the regular phosphorus diet, and the serum calcium level was regulated by other variables the most during consumption of the high-phosphorus diet. A sophisticated relationship exists between calcium and phosphorus homeostasis. Ba et al. found that the activation of the calcium-sensing receptor (CaSR) directly impedes PTH-sensitive phosphorus absorption by proximal tubules [44]. It has also been discovered that the FGF23/Klotho endocrine axis and the PTH/PTHR endocrine axis are the two fundamental pathways involved in phosphorus and calcium homeostasis [45]. These results indicate the essential role of serum calcium in the network of phosphorus metabolism during consumption of regular and high-phosphorus diets.

Consistently, using repeated measures correlation analysis, we found associations between 1,25 D and serum calcium levels, 1,25 D levels and urinary phosphorus excretion, PTH levels and urinary phosphorus excretion, and PTH and BALP levels during regular phosphorus diet intervention. We found associations between 1,25 D and serum calcium levels, serum phosphate levels and urinary phosphorus excretion, α-Klotho and FGF23 levels, and α-Klotho and 1,25 D levels during the low-phosphorus diet intervention. We found associations between serum calcium and FGF23 levels, 1,25 D levels and urinary phosphorus excretion, α-Klotho and FGF23 levels, and α-Klotho and PTH levels during the high-phosphorus diet intervention. However, the repeated measures correlation analysis revealed only individual relationships without exploring the interactions among these factors. It was unable to discover the most important variable and the role of each variable in a complex network. Moreover, it was incapable of inferring the sequence of each regulatory relationship between the phosphorus-associated regulatory factors (the cause and effect of the Granger causal relationship) we discussed above.

There are several limitations to our study. First, Granger causality only shows statistical causal relationships among variables. Further studies are needed to explore the causality of these variables. However, the Granger causality results provide new means to identify the main pathway of phosphorus metabolism, which could support further intervention. Second, only six participants were recruited due to the complexity and long duration of the study. However, as a phase I trial, a limited sample size is acceptable. There might be selection bias since we only included healthy young men. This also suggests that the findings of this study may not be extrapolated to phosphorus metabolism in the context of chronic kidney failure. Third, phosphate homeostasis is maintained by a delicate balance between intestinal absorption, renal excretion, and influx to and efflux from bone. Unfortunately, we did not include direct measurements of intestinal phosphate absorption and influx to and efflux from bone since it was difficult to measure these in the clinic. Although we included BALP levels as a measure related to bone, the measurements of influx to and efflux from bone cannot be estimated by the BALP level. Finally, there might be other variables that were not taken into account, such as 25-hydroxyvitamin D_3_, which suppresses PTH secretion independently of 1,25 D [46]. The Granger analysis needs to be improved as soon as new regulatory factors are identified.

In the present Granger causality analysis, variations in dietary phosphorus intake contributed to changes in the levels of central factors and causal relationships in the phosphorus metabolism network. The dominant factors in phosphorus metabolism following regular, low-, and high-phosphorus diets were α-Klotho, urinary phosphorus excretion, and 1,25 D and FGF23, respectively. The application of the Granger causality test and graph analysis sheds new light on the physiological regulatory mechanism of phosphorus metabolism, and it also provides new ways to investigate complex metabolic relations.

## Supplementary Material

Supplemental Material

Supplemental Material
