# Supplementary material for: Dominant factors of the phosphorus regulatory network differ under various dietary phosphate loads in healthy individuals
Source: Ren Fail. 2021 Jun 30;43(1):1076–86. doi: 10.1080/0886022X.2021.1945463 (PMC8253199; doi:10.1080/0886022X.2021.1945463)
Supplement: Supplemental Material [file IRNF_A_1945463_SM1960.pdf]

**Table 1. Baseline characteristics of six healthy participants.**

| Characteristics (Male, n=6) | Mean ± SEM |
|---|---|
| Age (year) | 28.5±2.1 |
| Weight (kg) | 64.5±2.4 |
| Height (cm) | 173.7±2.2 |
| Systolic pressure (mmHg) | 111.8±5.2 |
| Diastolic pressure (mmHg) | 75.3±3.1 |
| Body mass index (kg/m$^2$) | 21.4±0.5 |
| eGFR (ml/min per 1.73m$^2$) | 114.7±2.9 |
| Hemoglobin (g/l) | 141.8±4.5 |
| Serum albumin (g/l) | 47.0±0.9 |
| Blood biochemical indicators | |
| Glucose (mmol/l) | 4.8±0.2 |
| TG (mmol/l) | 0.6±0.1 |
| TC (mmol/l) | 4.0±0.3 |
| ALT (U/l) | 17.3±1.9 |
| AST (U/l) | 16.7±0.7 |
| BUN (mmol/l) | 4.0±0.3 |
| Serum creatinine (umol/l) | 78.3±2.5 |
| Uric acid (mmol/l) | 0.3±0.1 |
| Serum phosphate (mmol/l) | 1.1±0.1 |
| Serum calcium (mmol/l) | 2.1±0.1 |

*Note:* Data are shown as mean ± SEM or median (interquartile range). Abbreviations: eGFR, estimated glomerular filtration rate calculated by the EPI equation; TG, triglyceride; TC, total cholesterol; ALT, glutamic-pyruvic transaminase; AST, glutamic-oxalacetic transaminase; BUN, urea nitrogen; Serum calcium, serum corrected calcium. Conversion factors for units: triglycerides in mmol/l to mg/dl, 88.6; serum creatinine in umol/l to mg/dl, 0.01131; eGFR in ml/min/1.73m$^2$ to mL/s/1.73m$^2$, 0.01667; serum calcium in mmol/l to mg/dl, 4; serum phosphate in mmol/l to mg/dl, 3.1.

**Table 2. Repeated measures correlation analysis of each mineral metabolic variable following different types of phosphorus diets.**

Regular-phosphorus diet

| Independent \ Dependent | Serum Ca | Serum Pi | α-Klotho | FGF23 | BALP | PTH | $1,25(OH)_2D_3$ | Urinary Pi/Cr |
|---|---|---|---|---|---|---|---|---|
| Serum Ca | | 0.29 | 0.15 | 0.96 | 0.05 | 0.63 | 0.01 | 0.13 |
| Serum Pi | -0.15 | | 0.16 | 0.44 | 0.82 | 0.02 | 0.06 | 0.07 |
| α-Klotho | 0.21 | 0.20 | | 0.50 | 0.79 | 0.15 | 0.21 | 0.70 |
| FGF23 | -0.01 | 0.11 | -0.10 | | 0.83 | 0.39 | 0.51 | 0.07 |
| BALP | -0.28 | 0.03 | -0.04 | 0.03 | | <0.01 | 0.39 | 0.45 |
| PTH | 0.07 | 0.34 | 0.21 | 0.12 | 0.42 | | 0.06 | 0.03 |
| $1,25(OH)_2D_3$ | 0.38 | 0.27 | 0.18 | -0.10 | -0.13 | 0.27 | | <0.01 |
| Urinary Pi/Cr | 0.22 | 0.26 | 0.06 | 0.26 | 0.11 | 0.31 | 0.40 | |

Low-phosphorus diet

| Independent \ Dependent | Serum Ca | Serum Pi | α-Klotho | FGF23 | BALP | PTH | $1,25(OH)_2D_3$ | Urinary Pi/Cr |
|---|---|---|---|---|---|---|---|---|
| Serum Ca | | 0.98 | 0.53 | 0.94 | 0.50 | 0.36 | <0.001 | 0.16 |
| Serum Pi | -0.005 | | 0.13 | 0.07 | 0.81 | 0.29 | 0.37 | 0.04 |
| α-Klotho | 0.09 | 0.22 | | 0.04 | 0.68 | 0.05 | 0.04 | 0.83 |
| FGF23 | 0.01 | -0.26 | -0.30 | | 0.28 | 0.28 | 0.57 | 0.26 |
| BALP | 0.10 | -0.03 | 0.06 | -0.16 | | 0.29 | 0.61 | 0.38 |
| PTH | -0.13 | 0.16 | -0.28 | 0.16 | -0.16 | | 0.92 | 0.79 |
| $1,25(OH)_2D_3$ | -0.46 | -0.13 | -0.30 | 0.08 | 0.08 | 0.01 | | 0.67 |
| Urinary Pi/Cr | -0.20 | 0.30 | 0.03 | -0.17 | -0.13 | -0.04 | 0.06 | |

High-phosphorus diet

| Independent \ Dependent | Serum Ca | Serum Pi | α-Klotho | FGF23 | BALP | PTH | 1,25(OH)$_2$D$_3$ | Urinary Pi/Cr |
|---|---|---|---|---|---|---|---|---|
| Serum Ca | | 0.11 | 0.04 | 0.03 | 0.96 | 0.14 | 0.80 | 0.40 |
| Serum Pi | -0.23 | | 0.59 | 0.42 | 0.15 | 0.09 | 0.02 | 0.19 |
| α-Klotho | 0.29 | -0.08 | | $<$0.001 | 0.83 | $<$0.001 | 0.46 | 0.45 |
| FGF23 | -0.31 | 0.12 | -0.65 | | 0.49 | 0.20 | 0.72 | 0.66 |
| BALP | 0.01 | 0.21 | 0.03 | 0.10 | | 0.81 | 0.78 | 0.91 |
| PTH | -0.22 | 0.24 | -0.54 | 0.19 | 0.04 | | 0.10 | 0.47 |
| 1,25(OH)$_2$D$_3$ | 0.04 | -0.32 | -0.11 | 0.05 | 0.04 | -0.24 | | 0.03 |
| Urinary Pi/Cr | 0.12 | -0.19 | 0.11 | -0.06 | 0.02 | 0.10 | -0.32 | |

*Note:* Grey fill area: Repeated measures correlation coefficient between two metabolic variables; white fill area: *P*-value for the correlation of two metabolic variables. Abbreviations: Serum Ca, serum corrected calcium; Serum Pi, serum phosphate; FGF23, fibroblast growth factor 23; BALP, bone alkaline phosphatase; PTH, parathyroid hormone; Urinary Pi/Cr, urinary phosphorus/creatinine rate.

**Table 3. Graph theory and network analysis of the interactions among variables in phosphorus regulatory network following different dietary interventions.**

| | 1,25(OH)$_2$D$_3$ | Serum Pi | FGF23 | Serum Ca | Urinary Pi/Cr | α-Klotho | BALP | PTH |
|---|---|---|---|---|---|---|---|---|
| Regular-phosphorus diet | | | | | | | | |
| Betweenness | 0.07 | 0.03 | 0.12 | 0.14 | 0.13 | 0.22 | 0.01 | 0.12 |
| Out degree | 0.57 | 0.29 | 0.43 | 0.71 | 0.29 | 0.71 | 0.14 | 0.57 |
| In degree | 0.43 | 0.43 | 0.57 | 0.29 | 0.71 | 0.57 | 0.29 | 0.43 |
| Low-phosphorus diet | | | | | | | | |
| Betweenness | 0.15 | 0.15 | 0.15 | 0.02 | 0.22 | 0.04 | 0.08 | 0.01 |
| Out degree | 0.43 | 0.71 | 0.57 | 0.57 | 0.57 | 0.29 | 0.43 | 0.29 |
| In degree | 0.86 | 0.43 | 0.29 | 0.14 | 0.71 | 0.57 | 0.57 | 0.29 |
| High-phosphorus diet | | | | | | | | |
| Betweenness | 0.36 | 0 | 0.36 | 0 | 0 | 0 | 0 | 0.14 |
| Out degree | 0.29 | 0.14 | 0.57 | 0 | 0.14 | 0.14 | 0.29 | 0.29 |
| In degree | 0.43 | 0.14 | 0.29 | 0.43 | 0 | 0.29 | 0 | 0.29 |

Abbreviations: Serum Ca, serum corrected calcium; Serum Pi, serum phosphate; FGF23, fibroblast growth factor 23; BALP, bone alkaline phosphatase; PTH, parathyroid hormone; Urinary Pi/Cr, urinary phosphorus/creatinine rate.
