# Supplementary material for: Dominant factors of the phosphorus regulatory network differ under various dietary phosphate loads in healthy individuals
Source: Ren Fail. 2021 Jun 30;43(1):1076–86. doi: 10.1080/0886022X.2021.1945463 (PMC8253199; doi:10.1080/0886022X.2021.1945463)
Supplement: Supplemental Material [file IRNF_A_1945463_SM1957.pdf]

Supplements: one figure, three tables, overview of causal inference and explanations for panel Granger causality method

Supplementary Figure 1. Study design.

Supplementary Table S1. Diet composition of three types of intervention diet.

Supplementary Table S2. Nutritional status and mineral metabolism before diet interventions.

Supplementary Table S3. Changes in mineral metabolic variables of participants following different dietary interventions.

Supplementary Methods. Details and explanations for panel Granger causality and graph analysis method.

Supplementary Figure 1. Study design.

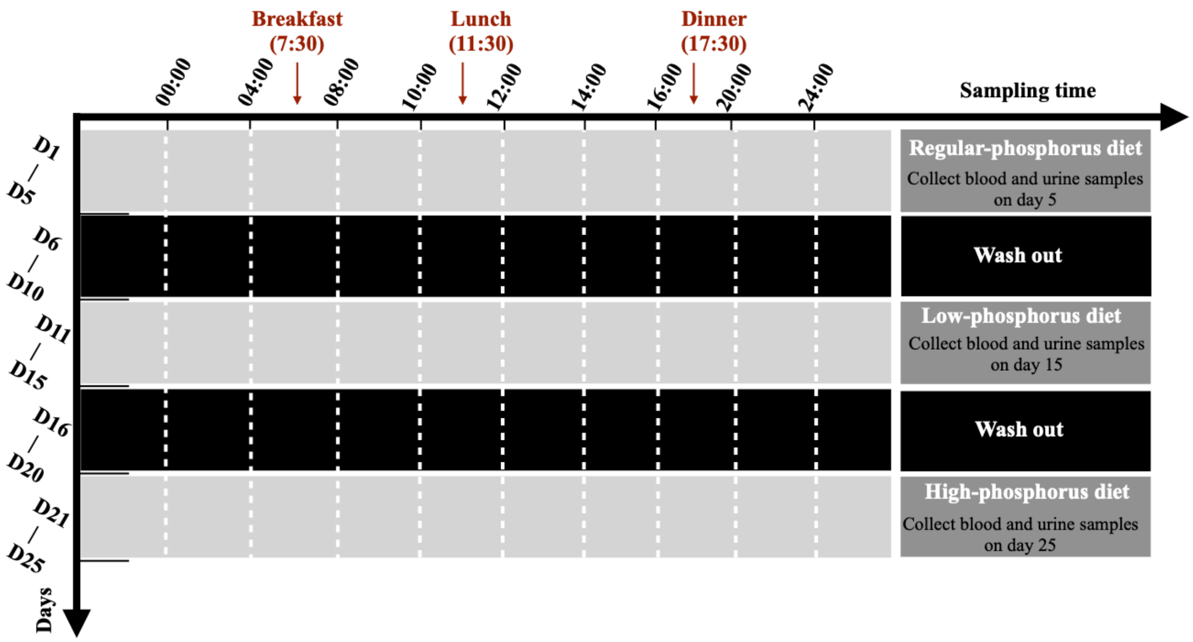

Supplementary Table S1. Diet composition of three types of intervention diet.

Diet	Calories (kcal)	Protein (g)	Phosphate (mg)	Calcium (mg)	Sodium (mg)
Regular-phosphorus diet	2177.0±22.6	112.4±1.3	1479.3±19.9	858.5±18.0	3006.8±86.1
Low-phosphorus diet	2154.1±6.5	50.6±1.2[a]	496.0±6.5[a]	794.7±35.5	2949.2±24.6
High-phosphorus diet	2172.2±23.6	110.4±1.8[b]	2221.1±10.7[ab]	779.0±47.6	3029.7±85.7

Note: Values are expressed as mean ± SEM. [a]$P<0.05$ vs Regular-phosphorus diet; [b]$P<0.05$ vs Low-phosphorus diet.

Supplementary Table S2. Nutritional status and mineral metabolism before diet interventions.

	Regular-phosphorus diet	Low-phosphorus diet	High-phosphorus diet
**Nutritional status**			
Weight (kg)	63.2±5.9	63.3±5.9	63.0±5.0
Serum albumin (g/l)	46.2 ±0.4	46.9±0.3	46.0±0.2
**Mineral metabolism**			
Serum phosphate (mmol/l)	1.4±0.1	1.5±0.1	1.5±0.1
Serum calcium (mmol/l)	2.2±0.1	2.2±0.1	2.2±0.1
Urinary Pi/Cr (mg/mg)	1.8±0.1	1.8±0.1	1.8±0.1
PTH (pg/ml)	34.0±2.1	34.4±2.0	34.2±2.4
FGF23 (pg/ml)	49.9±4.0	50.2±5.1	49.5±4.9
α-Klotho (pg/ml)	590.2±25.3	590.9±32.3	592.5±25.6
1,25$(OH)_2D_3$ (pmol/l)	83.5±4.0	84.4±5.9	85.2±3.3
BALP (ug/l)	0.9±0.1	0.8±0.1	0.8±0.1

Note: Values are expressed as mean ± SEM. All variables were measured **at 4:00 on the first day of each diet intervention**. Abbreviations: Serum calcium, serum corrected calcium; Urinary Pi/Cr, urinary phosphorus/creatinine rate; PTH, parathyroid hormone; FGF23, fibroblast growth factor 23; BALP, bone alkaline phosphatase. Conversion factors for units: albumin in g/L to g/dL, 0.1; serum calcium in mmol/l to mg/dl, 4; serum phosphate in mmol/l to mg/dl, 3.1.

Supplementary Table S3. Changes in mineral metabolic variables of participants following different dietary interventions.

	Regular-phosphorus diet	Low-phosphorus diet	High-phosphorus diet
Serum phosphate (mmol/l)	1.3±0.1	1.2±0.1[a]	1.5±0.1[ab]
Serum calcium (mmol/l)	2.2±0.1	2.3±0.1[a]	2.2±0.1
Urinary Pi/Cr (mg/mg)	2.2±0.1	1.1±0.1[a]	2.7±0.2[b]
PTH (pg/ml)	32.0±1.6	28.2±1.9	35.7±2.3
FGF23 (pg/ml)	49.6±1.7	49.2±2.1	53.6±2.7
α-Klotho (pg/ml)	602.3±12.9	581.3±13.2	574.7±15.4
$1,25(OH)_2D_3$ (pmol/l)	91.5±2.6	119.3±3.2[a]	94.3±2.4[b]
BALP (ug/l)	0.8±0.1	0.8±0.1	0.7±0.1[ab]

Note: Values are expressed as mean ± SEM. All variables were measured **at 9 time points on the fifth day of each diet intervention**. For each person, each variable was **the average value of the data collected in 9 time points**. Abbreviations: Serum calcium, serum corrected calcium; Urinary Pi/Cr, urinary phosphorus/creatinine rate; PTH, parathyroid hormone; FGF23, fibroblast growth factor 23; BALP, bone alkaline phosphatase. Conversion factors for units: serum calcium in mmol/l to mg/dl, 4; serum phosphate in mmol/l to mg/dl, 3.1. [a] P<0.05 vs regular-phosphorus diet; [b] P<0.05 vs low-phosphorus diet.

Supplementary Methods

Overview of Causal inference and explanations for panel Granger causality method

Causal inference is an important but not fully solved problem. In this section we give a brief review of Bayesian-based and Granger-based methods. The reader can find a throughout review of causal inference in S. Kleinberg's work [1].

Bayesian-based method, which establishes the casual network with a form of directed acyclic graph whose edges are calculated through the fitted probability distribution model. For a given state of variables, the Bayesian-based method can estimate the probability of the occurrences of the future event. The limitation of Bayesian-based method is that the strict presumed conditions can hardly be satisfied, and the model can be overfitted. Moreover, modify the Bayesian-based method to fit time-series data is difficult.

Granger-based method, which inferences the causality of time-series data with certain lagged time. Rather than fit a probability distribution model from the given dataset, the Granger-based method test the causal significance for individual relationship. Thus, Granger-based method is less prone to be overfitted. The limitation of the Granger-based method is that the causal significant relationship can be interpreted as a correlation of common variable. In this paper, we carefully control the selected variable to minimize the side-effect of the method.

Panel Granger-based method, which is an extension of Granger causality analysis that emphasizes the causality among multi-individual data involving measure variables. We prefer to the panel Granger causality method for three reasons.

(1) It is suitable for time-series data. The Bayesian-based methods cannot conveniently analysis time-series data as it must satisfy the assumptions of causal sufficiency, faithfulness, and causal Markov condition.

(2) It allows analyze panel data. The format of our experimental data are multi-individual data involving measurements over time. Thus, the data are naturally a panel shape.

(3) It supports to quantify causality. This feature enables us to exhibit the potential regulation relationships between variables via the weighted adjacency matrix.

The mathematical details of panel Granger causality method are elaborated as follows. We first organized the experimental data to panel form and make sure that all data were time-stationary which was verified via Levin, Lin and Chu (LLC) [3] panel data unit root test method. Then pairwise panel Granger causality analysis was performed to obtain the weighted adjacency matrix and construct the relationship graph between variables. Finally, we applied graph analysis to quantify causality.

A. Panel data and stationary analysis

A1. Panel data definition and construction

We constructed the collected time-series data as panel format, which is defined as equation (1).

$$\{x_{i,t}, i = 1,2, \dots, N, t = 1,2, \dots, T\} \qquad (1)$$

, where x is the investigated variable, i is the index of the sample, N is the total number of samples, t is the point of observed time, and T is the length of the observed time span. In our problem, the sample size N and time span T are fixed and comparable for each Pi consumption model, thus the data are categorized to short balanced panel. In this research, we focused on two dimensions of the panel data analysis. First case is the cross-sectional analysis, which addresses the impact on multiple variables on the single point of time. Second case is the time-series analysis, which addresses the samples variations across time span.

A2. Panel data unit root test

An important assumption for the Granger causality test [2] is that the investigated time-series data are stationary. We apply Levin, Lin and Chu (LLC) [3] panel data unit root test method to perform the stationary test. The autoregressive model is defined as equation (2).

$$x_{i,t} = \delta x_{i,t-1} + z'_{i,t}\gamma_i + \sum_{j=1}^{p_i} x_{i,t-1} + \varepsilon_{i,t} \qquad (2)$$

, where δ is the common root, $z'_{i,t}\gamma_i$ is the panel-specific means, p_i is the variable dependent lag order which can automatically defined via Akaike Information Criterion (AIC) [4] technique, $\varepsilon_{i,t}$ is the individual residual. Using equation (2), we can perform bias-adjusted t statistic with the following null hypothesis:

$$H_0: \delta = 0, \forall i, \dots, p_i \tag{3}$$

If H_0 is rejected, one can conclude that the panel data $x_{i,t}$ is stationary. We apply LLC method to all observed variables, the result (table x, appendix) showed that the panel data are stationary across all investigated variables.

B. Pairwise Panel Granger Causality

B1. Classic Granger causality test

The classic Granger causality[2] is defined as equation (4).

$$y_t = \sum_{k=1}^{p} \gamma^{(k)} y_{t-k} + \sum_{k=1}^{p} \beta^{(k)} x_{t-k} + \varepsilon_{x,t} \tag{4}$$

, where x_t and y_t are the under-test cause and effect variable, p is the time lag order, $\gamma^{(k)}$ and $\beta^{(k)}$ are the coefficients of the autoregressive model, $\varepsilon_{x,t}$ is variable's residual. F test statistic is performed with the following null hypothesis:

$$H_0: \beta^{(k)} = 0, \forall k = 1, \dots, p \tag{5}$$

If H_0 is rejected, one can conclude that the past of x_t information is helpful to explain y_t. Namely x_t and y_t have a causality relationship.

B2. Panel Granger causality test

The Dumitrescu-Hurlin (DH) test [5] extend classic Granger causality test to panel-wise data by equation (6).

$$y_{i,t} = \alpha_i + \sum_{k=1}^{p} \gamma_i^{(k)} y_{i,t-k} + \sum_{k=1}^{p} \beta_i^{(k)} x_{i,t-k} + \varepsilon_{i,t} \tag{6}$$

, where α_i is the individual effect which is constant over time, i is the index of the sample, other variables' meanings are consistent with equation (2). The null hypothesis and its alternative hypothesis for non-causality are therefore defined as follows.

$$H_0: \beta_i^{(k)} = 0, \forall k = 1, \dots, p, \forall i = 1, \dots, N \quad (7)$$

$$H_1: \beta_i^{(k)} = 0, \forall k = 1, \dots, p, \forall i = 1, \dots, N_1, \beta_i^{(k)} \neq 0, \forall k = 1, \dots, p, \forall i = N_1, \dots, N \quad (8)$$

, where N_1 is a variable range from 0 to N. If $N_1 = 0$, all individuals x_t are Granger causes for y_t On the other hand, if $N_1 = N$, no causal relationship between x_t and y_t for all individuals. DH method proposed an average Wald statistic $\overline{W}$ to measure the non-causality hypothesis deviation, which is defined as equation (9).

$$\overline{W} = \frac{1}{N} \sum_{i=1}^{N} W_i \quad (9)$$

, where W_i indicates the individual non-causality hypothesis deviation whose value larger or equal to 0. $W_i = 0$ implies to $\beta_i^{(k)} = 0$. Thus, we can use $\overline{W}$ to create causality graph's edges where larger weighted path indicates a stronger causal relationship. For a fixed timespan T $(T > 5 + 3p)$, we can test H_0 through p value of the standardized statistic $\tilde{Z}$ which is defined as equation (10)[5].

$$\tilde{Z} = \sqrt{\frac{N(T-3p-5)}{2p(T-2K-3)}} \left(\frac{T-3K-3}{T-3K-1} \overline{W} - p\right) \quad (10)$$

C. Graph analysis

C1. Graph construction

We defined a directed graph G associated with the granger causality as equation (11).

$$G = (V_G, E_G) \quad (11)$$

, where $V_G = \{x_1, x_2, \dots, x_m\}$ is the variable vertex, m is the total number of the investigated variables, $E_G = \{e_{12}, .., e_{ij}, .. e_{m(m-1)}\}, i \neq j$, is the edge. The weighted adjacency matrix for E_G is defined as equation (12).

$$M_{ij} = \overline{W}_{x_i \to x_j} \cdot I(p_{x_i \to x_j}) \quad (12)$$

, where $\bar{W}_{x_i \to x_j}$ is the average Wald statistics calculated from equation (9) which tests the causality for variables x_i and x_j. $I(p_{x_i \to x_j})$ is the incident function defined as equation (13), $p_{x_i \to x_j}$ is the P-value for $\tilde{z}_{x_i \to x_j}$ from equation (10) based on the standard normal distribution.

$$I\left(p_{x_i \to x_j}\right) = \begin{cases} 1, & p_{x_i \to x_j} < 0.05 \\ 0, & otherwise \end{cases} \quad (13)$$

C2. Graph measurements

Betweenness centrality, which measures the significance of the connectedness for a vertex. It is associated with the number of the shortest paths path through the specific vertex [6], which is defined as equation (14).

$$B(x_i) = \sum_{u,v \in V_G} \frac{\sigma(u,v|x_i)}{\sigma(u,v)} \quad (14)$$

, where $\sigma(u,v)$ is the total number of the shortest paths connecting (u,v), $\sigma(u,v|x_i)$ is the total number of the shortest paths connecting (u,v) via x_i .

In-degree centrality, which is the number of edges pointing to a vertex x_i divided by the number of all possible point-to paths. It is defined as equation (15).

$$InDegree(x_i) = \frac{\omega(G|x_i)}{m-1} \quad (15)$$

, where m is the number of vertexes, $\omega(G|x_i)$ is the number of incoming edges for x_i.

Outdegree centrality, which is the number of edges pointing out from a vertex x_i divided by the number of all possible point-out paths. It is defined as equation (16).

$$OutDegree(x_i) = \frac{\varphi(G|x_i)}{m-1} \quad (16)$$

, where $\varphi(G|x_i)$ is the number of outcoming edges for x_i.

Density, which measures the complexity of a given graph G. It is defined as the number of edges divided by the number of all possible connections as equation (17).

$$Density(G) = \frac{k}{m(m-1)} \quad (17)$$

, where k is the number of edges in G.

D. Method implementation

The panel data unit root check and granger causality were implemented via STATA packages *xtunitroot llc* [3] and *xtgranger* [7]. The graph construction and analysis were implemented by Python packages *Networkx* [8].

Reference:

1. Kleinberg S, Hripcsak G. A review of causal inference for biomedical informatics. J Biomed Inform. 2011; 44(6): 1102-1112.

2. Granger C W J. Investigating causal relations by econometric models and cross-spectral methods. Econometrica. 1969;424-438.

3. Levin A, Lin C F, Chu C S J. Unit root tests in panel data: asymptotic and finite-sample properties. J Econometrics. 2002;108(1): 1-24.

4.Yamaoka K, Nakagawa T, Uno T. Application of Akaike's information criterion (AIC) in the evaluation of linear pharmacokinetic equations. J Pharmacokinet Biopharm. 1978;6(2): 165-175.

5. Dumitrescu E I, Hurlin C. Testing for Granger non-causality in heterogeneous panels. Econ model. 2012; 29(4): 1450-1460.

6. Ulrik Brandes. A Faster Algorithm for Betweenness Centrality. J Math Sociol. 2001;25(2):163-177.

7. Lopez, L. and Weber, S. Testing for Granger causality in panel data. The Stata Journal. 2017;17(4): 972-984.

8. Hagberg A, Schult D, Swart P. Networkx: Python software for the analysis of networks. Mathematical Modeling and Analysis, Los Alamos National Laboratory, 2005.
